# High-Speed, UV-NIR Dual-Band Photodetection via a 2H-MoSe_2_/Si/1T-WS_2_ Bipolar Heterojunction

**DOI:** 10.3390/s26154949

**Published:** 2026-08-05

**Authors:** Zihao Wang, Meiping Tan, Lan Wang, Yan Xu, Yongqiang Yu

**Affiliations:** Micro Electromechanical System Research Center of Engineering and Technology of Anhui Province, Hefei University of Technology, Hefei 230009, China; 2023211817@mail.hfut.edu.cn (Z.W.);

**Keywords:** TMDCs, bipolar heterojunction, dual-band detection, high-speed, single-pixel imaging

## Abstract

Ultraviolet (UV) and near-infrared (NIR) dual-band photodetection is critical for applications ranging from secure optical communication and environmental monitoring to biomedical imaging. However, existing dual-band systems typically rely on discrete single-band detectors combined with optical filters, leading to complex alignment and high cost. Herein, we demonstrate a bipolar heterojunction (BHJ) based on a 2H-MoSe_2_/Si/1T-WS_2_ structure for filter-free, high-speed UV-NIR dual-band photodetection. The optimized device achieves high responsivities of 0.7 A/W@365 nm and 0.8 A/W@1064 nm at bias voltage of −2 V, along with a fast response time of 1.28 μs and a −3 dB bandwidth of 70 kHz. Furthermore, in comparative evaluations with broadband Si photodiodes, the BHJ successfully enabled high-quality NIR single-pixel imaging, reconstructing high-resolution images with 128 × 128 pixels under ambient lighting conditions. This work validates the potential of such transition metal dichalcogenides-Si heterojunctions for next-generation multispectral sensing and imaging systems.

## 1. Introduction

Ultraviolet (UV) and near-infrared (NIR) detection plays a vital role in numerous military and civilian applications, including space communications, environmental monitoring, and biomedical imaging [1,2,3]. The value of this dual-band approach lies in the complementary advantages of each region: NIR light is prized for its deep tissue penetration and low scattering, essential for night reconnaissance. Conversely, UV radiation operates with minimal background interference due to atmospheric absorption, offering high-contrast detection capability [4,5]. The integration of these complementary spectral bands within a single photodetector (PD) would substantially enhance the accuracy of target identification and tracking in complex environments [6]. However, current dual-band detection systems mainly use discrete single-band PDs with optical filters, which introduces significant challenges in optical alignment, greater system complexity, and increased cost [7,8]. To address these issues, the bipolar heterojunction (BHJ) structure offers a promising alternative. By design, it enables filter-free, dual-band operation: its monolithically integrated back-to-back junctions separate and collect photogenerated carriers from complementary spectral bands. This vertical architecture further ensures efficient carrier collection and a fast response [9,10,11]. However, most reported devices to date still exhibit modest responsivity, slow response speed, or limited ambient-light rejection, underscoring the pressing need for high-performance dual-band photodetectors capable of operating under practical conditions. The unique properties of two-dimensional transition metal dichalcogenides (TMDCs)—such as layer-dependent bandgaps and phase-dependent electronic structures—make them an excellent platform for high-performance BHJs [12]. In particular, 1T-WS_2_ offers metallic conductivity and strong NIR absorption, complementing the semiconductor properties of 2H-MoSe_2_, which is well-suited for UV detection [13,14]. Moreover, the absence of dangling bonds on TMDC surfaces allows van der Waals integration with Si without strict lattice matching requirements, facilitating the construction of 2D/3D heterojunctions that combine the advantages of TMDCs with the mature, low-cost Si technology. Recent advances—exemplified by the works of Pan et al., Xue et al., and Faella et al.—have demonstrated various strategies for dual-band or visible-suppressed detection [15,16,17]. However, these approaches typically rely on complex epitaxial growth or intricate material stacking, and often face trade-offs between spectral selectivity and response speed. In contrast, our work leverages a phase-engineered back-to-back heterojunction architecture to achieve monolithic, filter-free UV-NIR detection with ultrafast response and high ambient-light rejection.

In this work, we demonstrate a UV-NIR dual-band photodetector based on a 2H-MoSe_2_/Si/1T-WS_2_ BHJ fabricated via in situ PLD, with 1T-WS_2_ and 2H-MoSe_2_ sequentially deposited on opposite sides of an n-Si substrate. The MoSe_2_ thickness was optimized to confine the top depletion region near the surface for efficient UV harvesting, while the bottom Si/1T-WS_2_ Schottky junction is used for NIR detection. The resulting device achieves responsivities of 0.7 A/W@365 nm and 0.8 A/W@1064 nm, a response time of 1.28 μs, and a −3 dB bandwidth of 70 kHz. Furthermore, the BHJ enables high-quality NIR single-pixel imaging (SPI) (128 × 128 pixels) under ambient lighting, outperforming a commercial broadband Si photodiode by a factor of eight in SNR (4.0 dB vs. 0.5 dB). This work validates the potential of phase-engineered TMDC/Si heterojunctions for next-generation multispectral sensing and compact imaging systems.

## 2. Materials and Methods

*Device fabrication.* The n-Si substrates ((100), 1–10 Ω⋅cm, 500 μm, Nd = 1.5 × 10^15^ cm^−3^) were first cleaned by sequential ultrasonication in acetone, isopropanol, and DI water for 10 min each. Circular windows with a diameter of 5 mm were patterned on both sides of the substrate using high-temperature adhesive tape as a mask, defining an effective active area of approximately 19.6 mm^2^. The exposed SiO_2_ layer was then etched using BOE solution for 3–5 min. The 1T-WS_2_ and 2H-MoSe_2_ films were deposited in situ on opposite sides *via* pulsed laser deposition (PLD) (Lambda Physik COM-PexPro 102, 248 nm, Coherent Inc., Santa Clara, CA, USA) under optimized conditions: 1T-WS_2_ at 600 °C (3 Hz, 100 mJ, 20 min) and 2H-MoSe_2_ at 400 °C (2 Hz, 100 mJ, 10 min). After deposition, graphene films were wet-transferred onto both sides of the heterojunction as transparent electrodes using a PMMA-supported method. Silver paste was applied to the graphene electrodes to form external contacts.

*Device characterization.* The as-deposited 2H-MoSe_2_ and 1T-WS_2_ films were characterized by Raman spectrometer (LabRAM HR800, HORIBA Jobin Yvon, Pari, France), atomic force microscope (AFM, Dimension Icon, Bruker, Billerica, MA, USA) and X-ray photoelectron spectroscopy (XPS, ESCALAB 250Xi, Thermo Fisher Scientific, Waltham, MA, USA). The photoelectric properties of the BHJ were measured using a Keithley 4200-SCS semiconductor characterization system (Keithley Instruments, Cleveland, OH, USA) and laser diodes (Thorlabs, Newton, NJ, USA) with wavelengths of 254 nm, 365 nm, 405 nm, 520 nm, 650 nm, 780 nm, 808 nm, 980 nm, and 1064 nm. The response speed was evaluated with a system comprising a SIGLENT SDG5122 waveform generator (SIGLENT Technologies, Shenzhen, China) and a Tektronix TDS2022B oscilloscope (Tektronix, Beaverton, OR, USA). Specifically, the active device area was 19.6 mm^2^, and the illumination spot size is sufficiently large to fully cover the entire active area. The incident light intensity was calibrated using a Thorlabs PM100D optical power meter (Thorlabs, Newton, NJ, USA), and the corresponding optical power density (i.e., power per unit area) was calculated for the following responsivity calculations. The −3 dB bandwidth was measured via DC photovoltage response to sinusoidal laser modulation (980 nm, 100 Hz–1 MHz) using a waveform generator (SIGLENT SDG5122) and an oscilloscope (Tektronix TDS2022B).

*Imaging system.* The BHJ served as the single-pixel photodetector in our NIR SPI system. The setup included a digital micromirror device (DMD, DLP V-650L, Texas Instruments, Dallas, TX, USA), an 850 nm LED array (Thorlabs, Newton, NJ, USA), a data acquisition card (DAQ, NI 6351, National Instruments, Austin, TX, USA), a lens, and a target. For a fair comparison, a commercial Si photodiode (FDS100, Thorlabs, Newton, NJ, USA, 13.7 mm^2^, 0 V_bias_) was tested under identical optical path, power, patterns, and DAQ settings.

## 3. Results and Discussion

**Figure 1:**
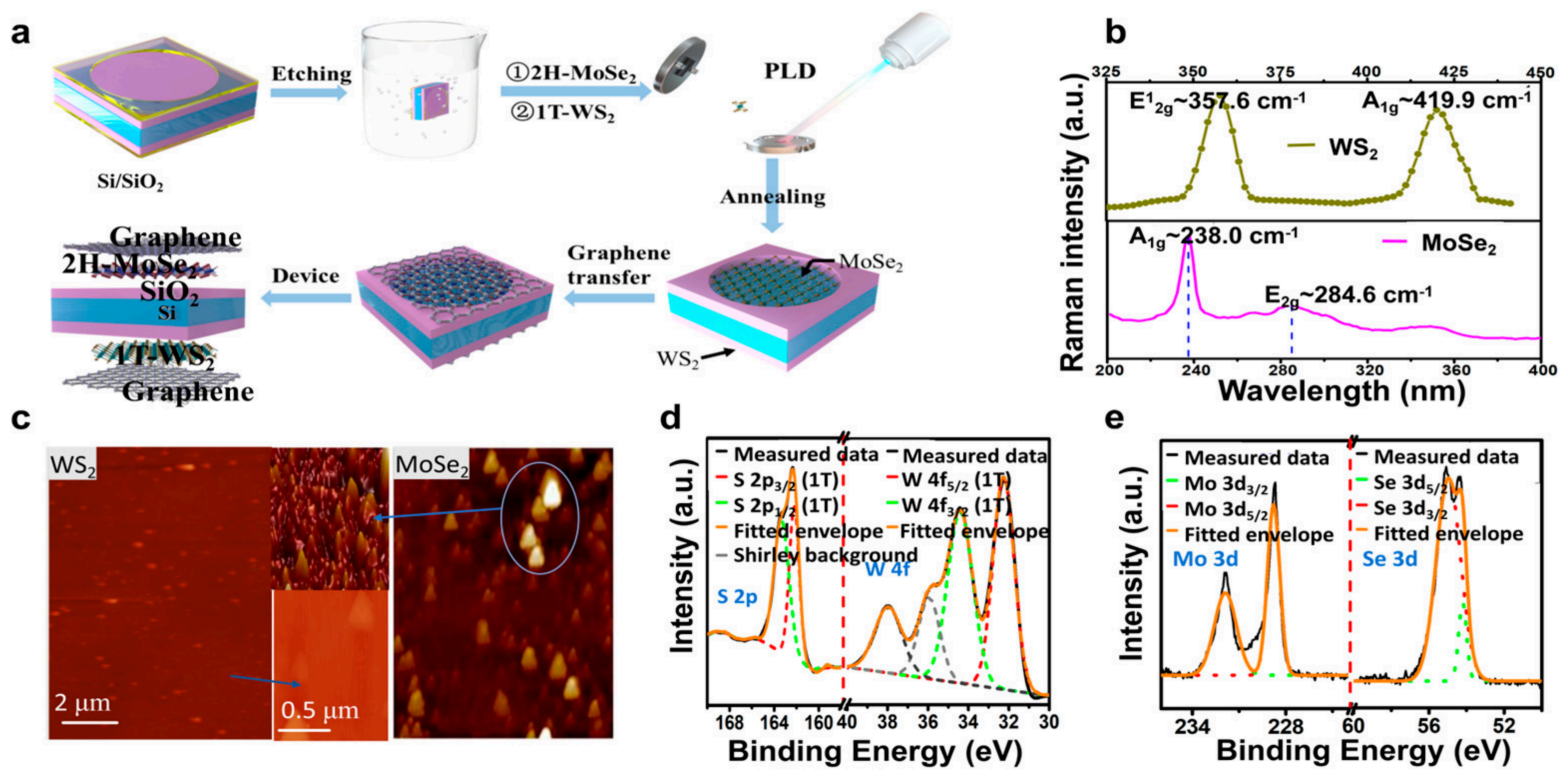
(**a**) Schematic illustration of the fabrication steps for 2H-MoSe_2_/n-Si/1T-WS_2_ BHJ-PD. (**b**) Raman spectra of 2H-Phase MoSe_2_ and 1T-Phase WS_2_ film. (**c**) AFM images of 2H-Phase MoSe_2_ and 1T-Phase WS_2_ nanosheets. Middle illustration: Partial magnified image for AFM. (**d**) The XPS spectra of the WS_2_ film. (**e**) The XPS spectra of the MoSe_2_ film.

As schematically depicted in Figure 1a, the 2H-MoSe_2_/Si/1T-WS_2_ BHJ was constructed by in situ depositing the 1T-WS_2_ and 2H-MoSe_2_ film onto both sides of a n-Si substrate, employing PLD technique. Raman spectroscopy (Figure 1b) confirmed the successful synthesis of 1T-WS_2_ and 2H-MoSe_2_, with characteristic peaks observed at 352 cm^−1^ and 419 cm^−1^ for 1T-WS_2_, and at 238 cm^−1^ and 284.6 cm^−1^ for 2H-MoSe_2_ [18,19]. AFM analysis revealed a flat surface morphology of the 1T-WS_2_ film, whereas the MoSe_2_ sample showed a vertically aligned triangular growth pattern (Figure 1c). Specifically, the root-mean-square (RMS) roughness is approximately 5 nm for the MoSe_2_ thin film and about 10 nm for the WS_2_ thin film, while the corresponding film thicknesses are 55 nm for MoSe_2_ and 75 nm for WS_2_. XPS measurement proved particularly effective for characterizing the structural phase of the WS_2_ film. As shown in Figure 1d, the W4f_7/2_ and W4f_5/2_ peaks are located at lower binding energies (~34.4 eV and ~32.2 eV, respectively) compared to those of the 2H phase (~33 eV and ~35 eV). This shift indicates a lower valence state of tungsten and confirms the predominant formation of the metallic 1T-phase structure [20,21]. The Mo and Se 3d orbitals account for the respective binding energies observed in the MoSe_2_ XPS spectrum [22]. The XPS peak-fitting deconvolution of the W4f spectrum (Figure 1d) reveals that the area ratio of the metallic 1T phase peaks represents approximately 88%, while the oxide impurity (W^6+^) and remnant 2H phase account for about 8% and less than 4%, respectively. This finding demonstrated that the PLD process provided a viable route for fabricating high-quality TMDCs films with controlled phase.

**Figure 2:**
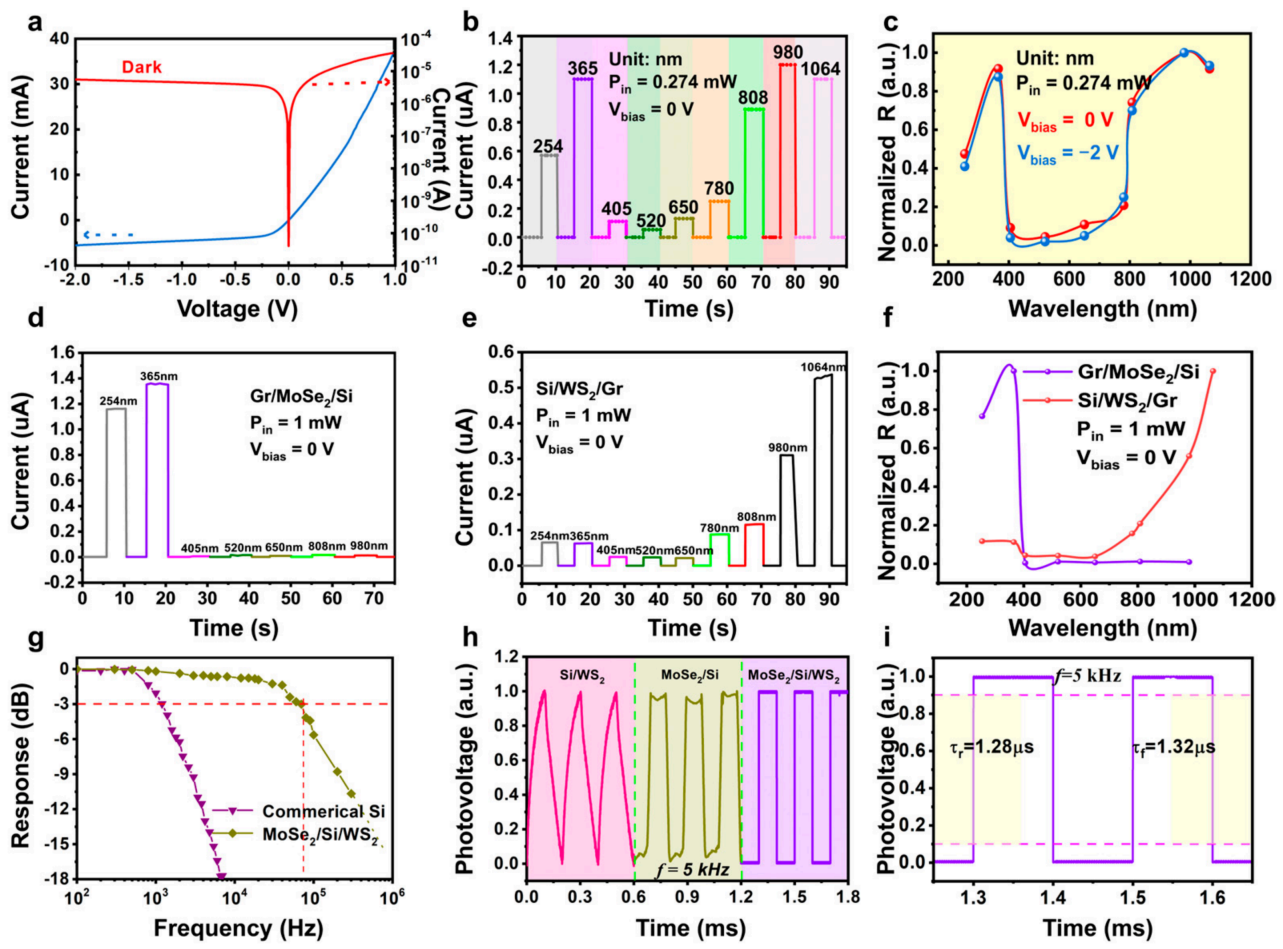
(**a**) Dark I-V characteristics of the BHJ-PD. (**b**) Time response of the BHJ-PD under multi-wavelength illumination at V_bias_ of 0 V. (**c**) Normalized spectral response of the BHJ-PD under multi-wavelength illumination at V_bias_ of 0 and −2 V, respectively. (**d**) Time response of the MoSe_2_/Si heterojunction. (**e**) Time response of the Si/WS_2_ heterojunction. (**f**) Normalized spectral response of MoSe_2_/Si and Si/WS_2_ under multi-wavelength illumination at V_bias_ of 0 V respectively. (**g**) Photoresponse characteristic of the BHJ-PD and commercial Si under 980 nm illumination. (**h**) Time response of Si/1T-WS_2_, 2H-MoSe_2_/Si and the BHJ at a pulsed light frequency of 5 kHz under 980 nm illumination. (**i**) Time response of the BHJ at a pulsed light frequency of 5 kHz under 980 nm illumination.

Figure 2a shows the I-V characteristic of the 2H-MoSe_2/_Si/1T-WS Confirmed. We have carefully reviewed Figure 1. We acknowledge that there is some overlapping content in panels (a), (d), and (e). However, we confirm that this overlapping does not affect the scientific understanding of the data or the overall clarity of the figure. Therefore, no revision is needed for these panels. BHJ-PD measured in the dark, indicating clear asymmetric rectification: under forward bias (positive voltage on the top electrode), the current rises rapidly to several milliamperes, while under reverse bias, the dark current remains extremely low (~5 × 10^−10^ A). The time response characteristics of the BHJ, measured under illumination at different wavelengths with the same optical power of 0.274 mW and at a bias voltage (V_bias_) of 0 V, are presented in Figure 2b. The I_photo_/I_dark_ ratio exceeds 10^4^ under both 365 nm and 1064 nm illumination, demonstrating excellent signal-to-noise performance, which is crucial for further applications in SPI. It is important to note that the corresponding normalized spectral response (Figure 2c) displays two distinct peaks at 365 nm and 980 nm at a V_bias_ of 0 V and −2 V, respectively, thereby confirming the dual-band, visible-blind detection capability. This unique spectral selectivity can be reasonably attributed to the spatial separation of the two depletion regions: the top MoSe_2_/Si junction primarily absorbs UV light due to its shallow penetration depth (Figure 2d), while the bottom Si/WS_2_ junction efficiently absorbs NIR light that can reach the deep interface (Figure 2e). Visible photons, with intermediate penetration depths, generate electron-hole pairs primarily in the neutral Si region where no built-in field exists, leading to rapid recombination and negligible photocurrent. The UV-NIR dual-band photoresponse of the 2H-MoSe_2_/Si/1T-WS_2_ BHJ can be plausibly interpreted as resulting from the synergistic effect of the two constituent heterojunctions (2H-MoSe_2_/Si and Si/1T-WS_2_), as supported by their respective photoresponse characterization (Figure 2d–f) and numerical simulations. We acknowledge that while the individual-junction measurements provide strong evidence for spectral assignment, the detailed carrier generation and collection pathways within the integrated BHJ are presented here as a simulation-supported interpretation rather than a fully experimentally verified mechanism.

Response speed and bandwidth are key metrics reflecting a PD’s ability to track fast optical signals. The normalized photoresponse V_DC_(f)/V_DC_(f_0_) (where f_0_ = 100 Hz is the low-frequency reference) was plotted as a function of the modulation frequency. Subsequently, the −3 dB bandwidth is defined as the frequency at which the normalized output voltage drops to 1/√2 (≈70.7%, corresponding to a −3 dB power drop) of its low-frequency reference value. From the experimental curve, this cut-off frequency was determined to be approximately 70 kHz (Figure 2g). Figure 2h shows the temporal response of individual junctions and the full BHJ at 5 kHz: the Si/1T-WS_2_ junction yields a slow, distorted waveform, the 2H-MoSe_2_/Si junction improves speed but remains suboptimal, while the complete BHJ produces a clean, well-defined square wave. Figure 2i further resolves the BHJ’s response, revealing nearly ideal rectangular pulses with a rise time (t_r_) of 1.28 μs and a fall time (t_f_) of 1.3 μs.

**Figure 3:**
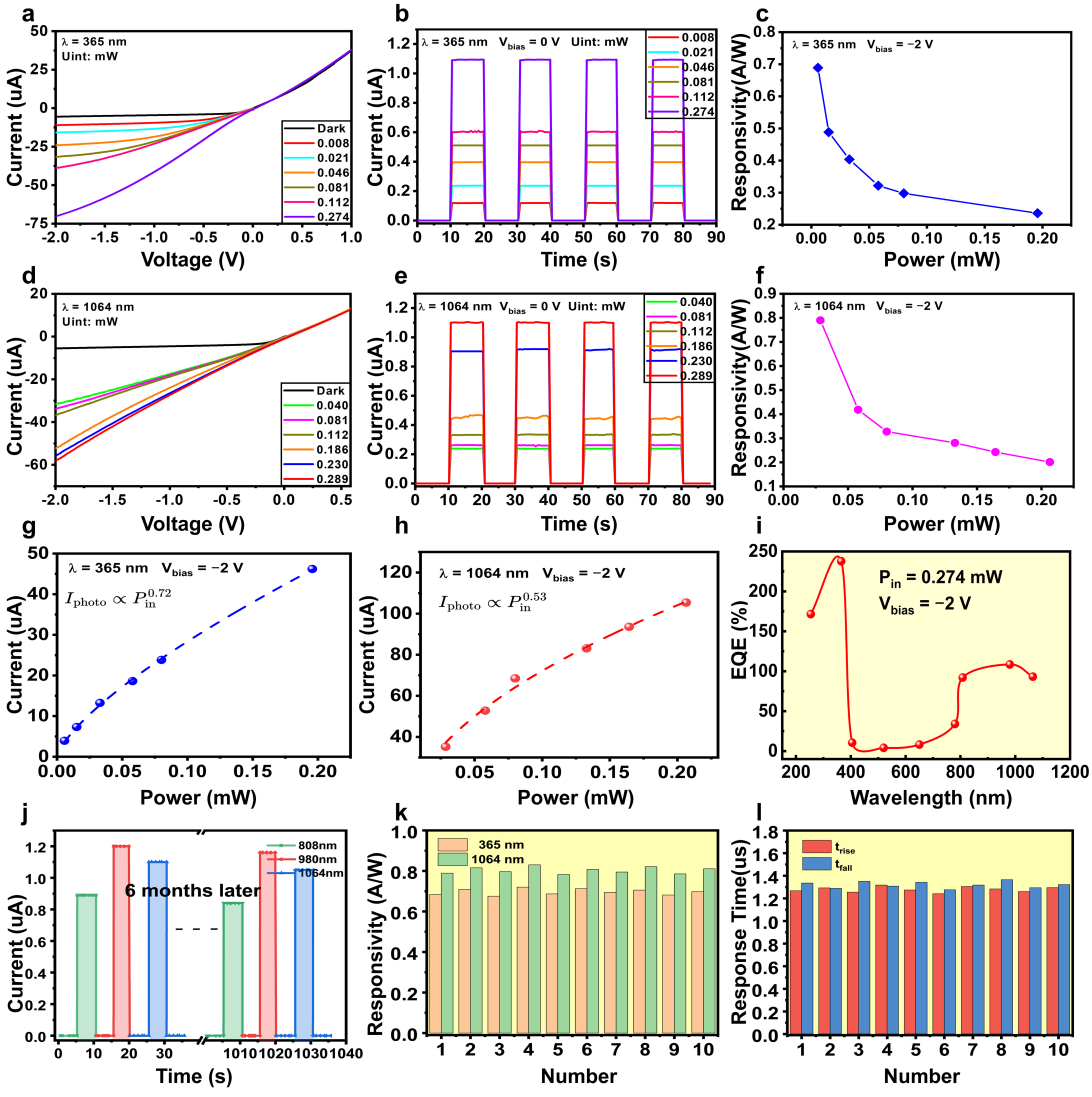
(**a**) I-V curves at 365 nm at various power. (**b**) Temporal photoresponse at 365 nm at Vbias of 0 V. (**c**) Power dependent responsivity curve for 365 nm at V_bias_ of −2 V. (**d**) I-V curves under 1064 nm illumination. (**e**) Temporal photoresponse under 1064 nm illumination at V_bias_ of 0 V. (**f**) Power dependent responsivity curve for 1064 nm at V_bias_ of −2 V. (**g**) The power-dependent photocurrent under 365 nm illumination. (**h**) The power-dependent photocurrent under 1064 nm illumination. (**i**) The external quantum efficiency (EQE) curve of the BHJ at V_bias_ of −2 V. (**j**) Time response of the BHJ at 0 V under NIR illumination before and after 6 months later. (**k**) Responsivity of 10 devices. (**l**) Response time of 10 devices.

To further evaluate the photoresponse in the target UV-NIR bands, we measured the I-V characteristics under varying optical power at 365 nm and 1064 nm (Figure 3a,d): the photocurrent scales remain consistent with incident power and increase under reverse bias, reflecting enhanced depletion width and more efficient carrier collection. The temporal response at V_bias_ = −2 V (Figure 3b,e) shows rapid, stable on/off switching with no persistent photoconductivity. This absence of PPC is highly consistent with our previous works on MoSe_2_/Si [14] and WS_2_/Si [19] heterojunctions, further confirming the reliable interface quality. Figure 3c,f gives the responsivity as a function of optical power, defined as

(1) $$R=\frac{I_{photo}}{P_{in}}$$

where *I_photo_* is the photocurrent and *P_in_* is the incident optical power. The R of the PD decreases as the *P_in_* increases. This is because with the increase in incident light power, the light absorption capacity of the material gradually tends to saturate. The BHJ achieves peak responsivities of 0.7 A/W@365 nm (Figure 3c) and 0.8 A/W@1064 nm (Figure 3f); the decrease in R at higher powers reflects gradual saturation of light absorption and increase in the recombination rate. Notably, the 0.7 A/W responsivity at 365 nm corresponds to a photoconductive gain of ~2.4. While deep-level traps typically degrade response speed, we speculate that this moderate gain may be hypothetically associated with shallow-level defect states, such as potentially ambient-induced Se vacancy-oxygen complexes (V_Se_ + O_2_) at the vertically aligned MoSe_2_ grains. Recent ultrafast spectroscopic studies indicate that carrier trapping and release dynamics at such shallow traps occur on a sub-picosecond timescale [23]. The absence of persistent photoconductivity (PPC) indicates that the density of deep-level interface traps is negligibly low; otherwise, prolonged carrier trapping would manifest as PPC tails and severely degraded speed. The metallic 1T-WS_2_ contact partially alleviates Fermi-level pinning via electrostatic screening of interface dipoles, yet the functional integrity of both junction barriers is confirmed by the rectification (Figure 2a) and dual-band spectral response (Figure 2c). This ultrafast detrapping is orders of magnitude faster than our device’s overall RC-limited response (~1.28 μs), which could theoretically offer a scenario where the observed gain does not compromise the fast operation. We emphasize that this is merely a speculative interpretation based on literature reports, and direct experimental validation of these trap energy levels is beyond the scope of the current work and will be a key focus of our future research.

Figure 3g,h shows the power-dependent photocurrent under 365 nm and 1064 nm illumination, respectively. The photocurrent exhibits a nearly linear increase with increasing optical power density, indicating a strong dependence on incident light intensity. To quantitatively describe this relationship, we fitted the experimental data using the power law:

(2) $$I_{ph}=AP^{\alpha }$$

where I_ph_ is the photocurrent, A is a scaling constant (wavelength-dependent), P is the incident optical power, and α is the exponent that characterizes the photocurrent response to light intensity. The fitting yields α ≈ 0.72 and 0.53 for 365 nm and 1064 nm incident light at a bias of −2 V, respectively, suggesting efficient photogenerated carrier collection and superior photocurrent capability. The non-unity exponent is attributed to the complex processes of electron-hole generation, trapping, and recombination within the MoSe_2_, WS_2_ film and interface. Accordingly, the external quantum efficiency (EQE) was estimated as using the following formula:

(3) $$EQE=R\cdot \frac{hc}{q\lambda }$$

where *h*, *c*, *λ* and *q* are Planck’s constant, speed of light, light wavelength and electron charge, respectively. Figure 3i shows the wavelength dependent EQE curve of the BHJ at 0 V, demonstrating a strong and direct experimental evidence of the “visible-blind” dual-band detection capability of our device. To further substantiate the long-term stability, we extended the observation period to six months and recorded the time-dependent photoresponse at 808 nm, 980 nm and 1064 nm (Figure 3j). The measured results show that the photoresponse decay is less than 20% relative to the initial values, indicating satisfactory device durability. Since any significant phase transition from 1T to 2H would be expected to cause marked changes in the electrical and photoresponse characteristics, the sustained device performance over six months provides strong indirect evidence for the phase stability of the deposited 1T-WS2 film under our experimental conditions. Statistical analysis of responsivity and response time across ten devices confirms the high uniformity of the PLD-grown TMDC films and the reliability of our vertical heterojunction fabrication process (Figure 3k,l).

**Figure 4:**
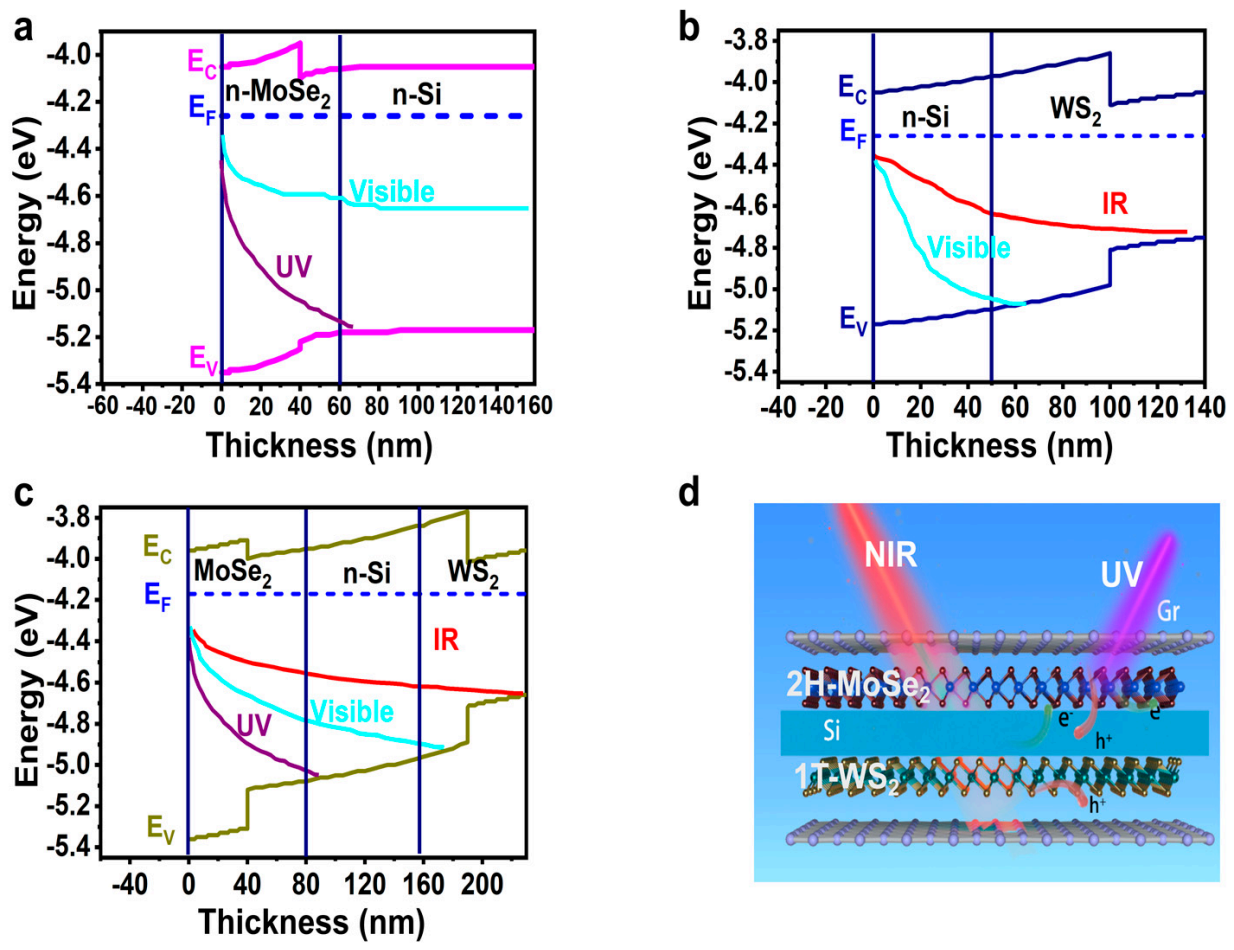
(**a**) Simulated band diagrams of MoSe_2_/Si heterojunctions under different wavelengths. (**b**) Simulated band diagrams of Si/WS2 heterojunctions under different wavelengths. (**c**) Energy band diagram of the full heterojunction under UV-Vis-NIR illumination. (**d**) Schematic diagram of carrier transport in the BHJ under illumination.

To elucidate the wavelength-selective photoresponse of the 2H-MoSe_2_/Si/1T-WS_2_ BHJ, we performed numerical simulations of the energy band diagrams using AFORS-HET (version 2.6, Helmholtz-Zentrum Berlin, Berlin, Germany). Figure 4a shows the simulated band diagram of the 2H-MoSe_2_/Si heterojunction with a MoSe_2_ thickness of 60 nm. It is evident that, owing to the presence of a thin MoSe_2_ layer, the depletion region is situated in close proximity to the top surface. It has been established that UV photons (which exhibit a shallow penetration depth) are completely absorbed within the surface depletion region, thereby generating electron-hole pairs that are efficiently separated by the built-in electric field. In contrast, visible and NIR photons, which have longer penetration depths, can pass through the 2H-MoSe_2_/Si junction without being absorbed. This phenomenon provides a rationale for the selective response of the top junction to UV light. For the bottom junction, Figure 4b shows the simulated band diagrams of the Si/1T-WS_2_ heterojunction. The 1T-WS_2_ layer exhibits metallic character (confirmed by Raman and XPS in Figure 1), forming a Schottky junction with n-Si. Under NIR illumination (1064 nm), photons penetrate through the entire Si layer and are absorbed near the Si/1T-WS_2_ interface. The photogenerated electrons in Si are collected by the n-Si side, while holes are injected into the metallic WS_2_ electrode. For UV and visible light, absorption occurs primarily in the top MoSe_2_ layer or near the Si surface, leaving little optical intensity reaching the bottom Si/1T-WS_2_ junction. Consequently, the Si/1T-WS_2_ junction is NIR-selective.

Figure 4c combines the two junctions into the full 2H-MoSe_2_/Si/1T-WS_2_ BHJ band diagram under UV-Vis-NIR illumination. Two depletion regions, distinctly separated in space, have been observed. The first depletion region (2H-MoSe_2_/Si) absorbs UV light, while the second depletion region (Si/1T-WS_2_) absorbs NIR light. The absorption and subsequent re-emission of visible light occurs predominantly within the intermediate Si bulk, with minimal contribution to the photocurrent. The band engineering in question is pivotal in facilitating the creation of a filter-free, dual-band (UV and NIR) PD, which is notable for its intrinsic visible rejection. Figure 4d provides a schematic representation of the photoinduced carrier separation and transport pathways. When exposed to UV illumination (365 nm), electron-hole pairs are generated in the 2H-MoSe_2_/Si depletion region. These pairs are separated by the built-in electric field, with the electrons drifting towards the n-Si side and the holes collected by the top graphene electrode. Under NIR illumination (1064 nm), the Si/1T-WS_2_ junction produces photogenerated electrons that are collected by the n-Si layer, and holes that are injected into the metallic 1T-WS_2_ and subsequently extracted by the bottom graphene electrode.

**Figure 5:**
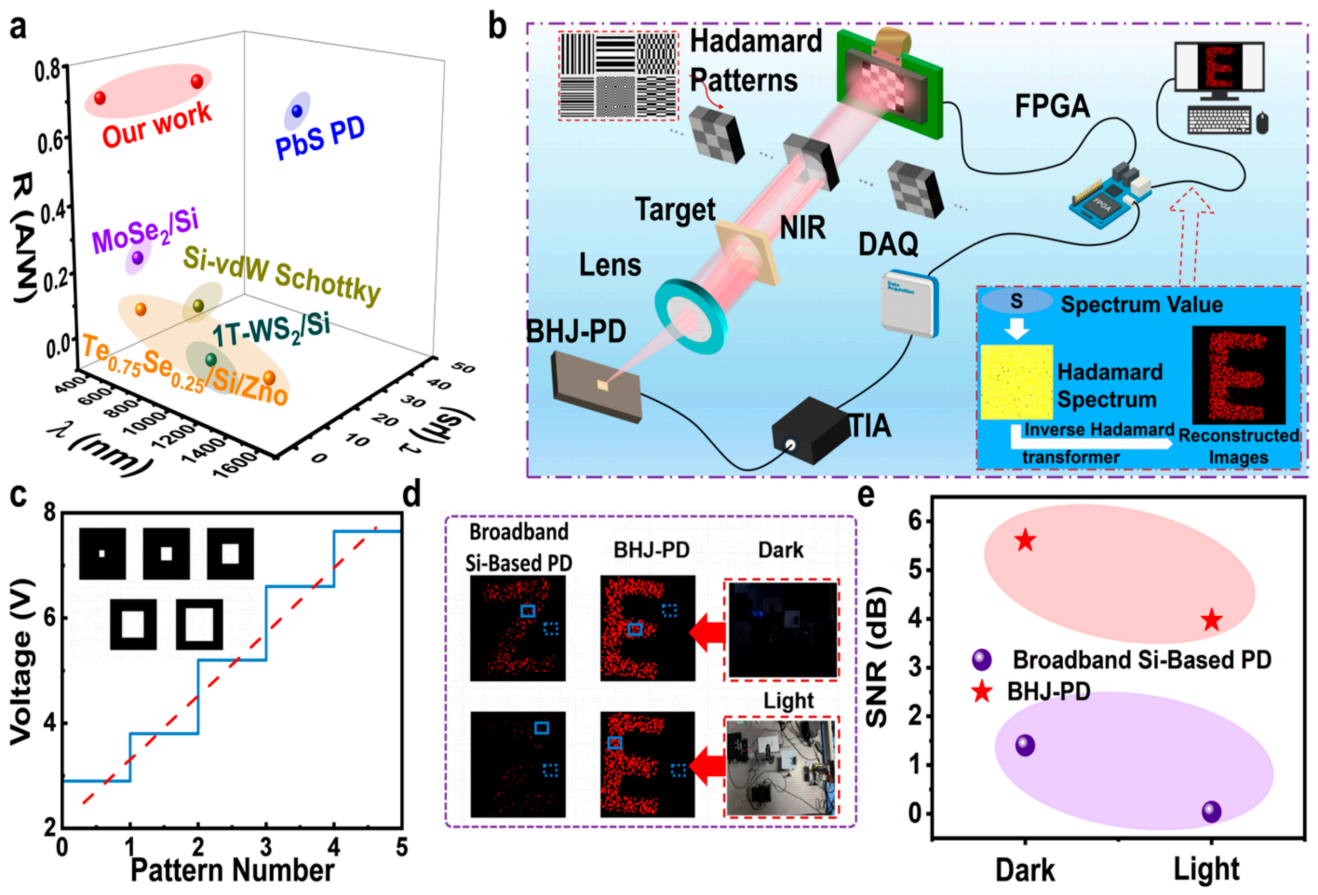
(**a**) Performance comparison of our device with other reported PDs, including PbS PD [24], MoSe_2_/Si [14], Si-vdW Schottky [25], 1T-WS_2_/Si [19], and Te_0.75_Se_0.25_/Si/ZnO [5]. (**b**) Schematic of the Hadamard NIR single-pixel imaging system. (**c**) The photovoltage acquisition curve of the BHJ-PD for five testing patterns. (**d**) Reconstructed imaging results under dark and ambient light conditions for the conventional broadband Si-based PD and the BHJ-PD, respectively. (**e**) Signal-to-noise ratio (SNR) comparison of the imaging results.

To rigorously benchmark the performance of our BHJ photodetector, Table 1 summarizes key metrics in comparison with recent state-of-the-art dual-band and visible-suppressed photodetectors. It should be noted that the specific detectivity (D*) for our device is denoted as “–” in Table 1. Due to the current unavailability of an ultra-low-noise current preamplifier and a spectrum analyzer for direct measurement of the noise current power spectral density (*S_I_*), we deliberately omitted the D* estimation based on the shot-noise approximation. Such an approximation would neglect *1*/*f* and generation-recombination noise, inevitably leading to a significant overestimation of the actual detectivity. To ensure scientific rigor, we instead emphasize the device’s excellent and directly measurable signal-to-noise ratio (evidenced by an *I_photo_*/*I_dark_* ratio exceeding 10^4^) and its robust imaging capability, which are more representative of its practical application potential. This comparison highlights the practical applicability of our device. Its unique UV-NIR dual-band photodetection capability, combined with high responsivity and high response speed as shown in Figure 5a [5,14,19,24,25], makes it a highly promising candidate for single-pixel photodetectors. Figure 5b provides the schematic photograph of the Hadamard single-pixel imaging (HSI) system, utilizing a DMD to project encoded Hadamard patterns onto the target at 4000 patterns/second [24]. The encoded Hadamard patterns were defined on the basis on the Hadamard transform as follows [26,27],

(4) $$\tilde{I_{H}}\left(u,v\right)=\frac{1}{MN}\sum_{x=0}^{M-1}\sum_{y=0}^{N-1}I\left(x,y\right){\left(-1\right)}^{\sum_{i=0}^{N-1}\left[b_{i}\left(x\right)b_{i}\left(u\right)+b_{i}\left(y\right)b_{i}\left(v\right)\right]}$$

where (*x*, *y*) are the spatial domain coordinates, (*u*, *v*) are the Hadamard domain coordinates, and *b_i_*(*z*) is the ith bit in the binary representation of the integer *z*. The NIR light (850 nm LED array) that has been transmitted or reflected is collected by a lens and focused onto the BHJ-PD. The photovoltage signal from the BHJ is recorded by a data acquisition card (DAQ) and subsequently processed by an inverse Hadamard transform to reconstruct the image. The high response speed enables it not only to discriminate signals across all tested projection speeds but also to sequentially convert the total transmitted light intensity into voltage signals. Moreover, the linear photovoltage response to the five test patterns, ranging from the lowest to the highest light power, indicates that the BHJ can operate effectively within the linear regime for NIR HSI, thereby enabling imaging capability (Figure 5c). To benchmark the reconstructed image quality, a broadband Si photodiode was used for high-resolution (128 × 128 pixels) imaging under both dark and ambient light conditions. Figure 5d displays the 128 × 128 reconstructed images obtained from the conventional broadband Si-based PD and the BHJ. In our SPI system, both PDs successfully reconstruct the letters “E” and “Z” under dark conditions. Under ambient light, however, the “Z” recovered by the broadband Si-based PD appears less distinct, whereas the BHJ maintains clear reconstruction. This result indicates that the BHJ can effectively suppress visible light interference, owing to its unique dual-band response. In addition, the signal-to-noise ratio (SNR) of these images was used as an evaluation metric, which is calculated as follows [25,28],

(5) $$SNR=\frac{\left\langle I_{f}-I_{b}\right\rangle }{(\sigma_{f}+\sigma_{b})/2}$$

where *I_f_* and *I_b_* are the mean intensities of the image features and the background. σ*_f_* and σ_b_ are the noise levels, which are the standard deviations of the intensities in the features and the background, respectively. Calculated SNR values for the BHJ were 5.6 dB (dark conditions) and 4.0 dB (ambient light conditions), whereas the broadband PD yielded only 1.5 dB and 0.5 dB under the same respective conditions, highlighting the BHJ’s superior signal fidelity (Figure 5e). We acknowledge that imaging at the device’s peak response wavelengths (e.g., 980 nm or 1064 nm) would be ideal for demonstrating optimal performance. However, we believe that the successful imaging at 850 nm—where the responsivity is lower—provides a conservative yet convincing proof of concept for the practical applicability of our BHJ-PD in NIR imaging systems. Taken together, the findings confirm that the BHJ-PD serves as a more promising candidate than the broadband PD for practical NIR SPI applications.

## 4. Conclusions

In summary, we developed a 2H-MoSe_2_/Si/1T-WS_2_ BHJ via in situ PLD, achieving filter-free, high-speed UV-NIR dual-band photodetection. The optimized BHJ-PD exhibits high responsivities of 0.7 A/W@365 nm and 0.8 A/W@1064 nm under V_bias_ of −2V, along with a fast response time of 1.28 μs and a −3 dB bandwidth of 70 kHz. Benefiting from its intrinsic visible-light rejection, the BHJ enables high-quality NIR SPI (128 × 128 pixels), even under ambient light, demonstrating clear advantage over traditional broadband Si photodetector. This work validates the potential of phase-engineered transition metal dichalcogenide/Si heterojunctions for next-generation multispectral sensing and compact imaging systems, paving the way for its practical implementation in single-pixel imaging systems operating under ambient lighting interference.

## Figures and Tables

**Table 1:** Performance comparison of our device with other reported PDs.

Device Structure	Detection Bands	*R* (A/W)	D* (Jones)	τ_r_/τ_f_ (μs)	Bandwidth (kHz)	Bias (V)	Ref.
2H-MoSe_2_/Si/1T-WS_2_ (This work)	365 nm 1064 nm	0.7/0.8	–	1.28/1.30	70	−2	-
Te_0.75_Se_0.25_/Si/AZO	638 nm 1550 nm	0.11/0.063	2.5 × 10^11^ 1.4 × 10^11^	1.32/1.42	260/280	0/±0.6	[5]
PbS CQDs/Si	850 nm	0.60	5.6 × 10^13^	43/72	52	0	[24]
WS_2_/SiHMs	700–1550 nm	0.251	–	4.7/7.96	50	0/−2	[19]
Mo_4/3_B_2-_XTZ/Si	350–1310 nm	0.0998	4.3 × 10^14^	11.8	23	0/−2	[25]
Gr/MoSe_2_/Si	365–1310 nm	0.270@650 nm	7.1 × 10^10^	0.27/0.35	~1000	0/−5	[14]

## Data Availability

The original contributions presented in this study are included in the article. Further inquiries can be directed to the corresponding author.
